# Comparison of the effect of teaching games for understanding, sport education, combined and linear pedagogy on motor proficiency of children with developmental coordination disorder

**DOI:** 10.3389/fpsyg.2024.1385289

**Published:** 2024-05-28

**Authors:** Behrouz Ghorbanzadeh, Sadettin Kirazci, Georgian Badicu

**Affiliations:** ^1^Department of Physical Education and Sport Sciences, Faculty of Education and Psychology, Azarbaijan Shahid Madani University, Tabriz, Iran; ^2^Department of Physical Education and Sport, Faculty of Education, Middle East Technical University, Ankara, Türkiye; ^3^Department of Physical Education and Special Motricity, Faculty of Physical Education and Mountain Sports, Transilvania University of Brașov, Braşove, Romania

**Keywords:** developmental coordination disorder, linear method, sports education method, teaching games for understand method, combined method, motor skills

## Abstract

**Introduction:**

The prevalence of developmental coordination disorder (DCD) is increasing and it has been shown that the main problem of children with DCD is their low motor proficiency. Therefore, it is important to find a way to improve motor skills in these children. Thus, this study aimed to compare the effect of teaching games for understanding (TGFU), sport education (SE), combined (TGFU and SE), and linear pedagogy (LP) on motor proficiency of children with DCD.

**Methods:**

In this regard, among 7-year-old children in Turkey, 80 children were selected voluntarily and by evaluating the MABCD-2 test. These children were randomly placed in four-LP (control), SE, TGFU, and combined (SE-TGFU) groups and practiced futsal exercises for 16 sessions under the supervision of coaches specific to each method. BOT-2 short-form test was used to evaluate motor proficiency.

**Results:**

The results of the analysis of the covariance test showed that the group effect is significant, and the results of the *post hoc* LSD test showed a significant difference between the LP with SE, LP with TGFU, LP with combination, SE with TGFU, SE with combination and TGFU with combination groups (*p* = <0.001).

**Discussion:**

Based on the results of this study, the combined method is the best compared to other methods. Generally, combining games as an important activity in childhood with SE that emphasizes improving the child’s self-esteem is a method that can solve the movement competence that is the main problem of DCD children and lead them to continue physical activity.

## 1 Introduction

Some children perform poorly in motor skills and daily activities compared to their peers; based on the Diagnostic Statistical Manual (DSM) of American Psychiatric Association (2013), this problem is called developmental coordination disorder (DCD) (Vahia, 2013). According to DSM criteria, these children’s motor competence is lower than children without the disorder, and it is difficult for them to enjoy physical activity because they do not believe in their abilities (Hendrix et al., 2014). Children with DCD are not independent in their work and avoid physical activity (Lloyd et al., 2006). Although DCD receives less attention than other developmental disorders, its impact can be severe and long-lasting (Hill and Barnett, 2019). This issue is important because DCD is recognized as a neurodevelopmental disorder based on the latest report of the DSM-5 (DSM-5) (Hill and Barnett, 2019).

International estimates show that the prevalence of this disorder is about 6% of school children with an emphasis on boys (American Psychiatric Association, 2013). Studies in England, Germany, the Netherlands, and Canada indicate a prevalence of 2.5–7.7% of DCD in children (Lingam et al., 2010; Blank et al., 2011; Baghernia and Mohammadizadeh, 2012; American Psychiatric Association, 2013). The prevalence of this disorder in Turkey boys is high, which is twice the ratio compared to girls (Tunçtürk et al., 2019). Children with this disorder have characteristics such as delay in the development of motor skills, inability to perform school activities and daily life, and finally, inability to perform sports and motor proficiency (Kirby and Sugden, 2007).

For the age group of 7–10 years, movement is a practical way to achieve complex motor skills, professional sports skills, and daily life. This period is one of the most important periods of life in consolidating movement, fundamental, and manipulation skills (Ozmun and Gallahue, 2016). However, for children with DCD, performing normal daily activities and school, especially motor proficiency, is a major challenge (American Psychiatric Association, 2013; Bieber et al., 2016). DCD children’s biggest problem is the low score of motor proficiency (American Psychiatric Association, 2013). Low motor proficiency is related to self-esteem and physical activity eventually leads to obesity and overweight, which endangers the child’s health in the future (Stodden et al., 2008; Ozmun and Gallahue, 2016). Therefore, it is important to try to improve the motor proficiency of DCD children. It has been shown that motor interventions have been a successful way to improve the motor proficiency of children with DCD (Crova et al., 2014; Farhat et al., 2015). However, the main issue in children with movement disorders is how to teach movement interventions (Ebrahimi Tavakolian et al., 2020). In this regard, games can be used because children enjoy it (Butler and McCahan, 2005).

One of the games is teaching games for understanding (TGfU), which is a representation of a real game with simpler rules (Butler and Griffin, 2010). TGfU is a new model introduced by Bunker and Thorpe as an alternative to the traditional skill-based approach to teaching sports skills; besides, TGFU has attracted the attention of many teachers, educators, and researchers (Werner et al., 1996). Game-based approaches such as TGFU introduce simple game tactics first, and skill practice comes next upon demand. In the TGFU approach, what should be done comes first, and the necessary training is provided before the way to do it (Tan, 2018). In this method, communication between tactics and techniques that aim to promote intelligent and skillful performance is suggested (Ríos et al., 2019). This type of game can be very effective for creating excitement in children and motivating them to continue the activity by raising self-esteem and motor competence (Ríos et al., 2019).

There is another model in physical education teaching, known as sports education (SE). The SE model aims to create competent and enthusiastic students (Siedentop et al., 2011). According to Kirk (2013), it is an evidence-based instructional model in which teachers focus on student learning facilitated through constructivist instruction through six features: (1) goals are organized, (2) children become members of teams to have a commitment to the team, (3) in the form of a game, competition between teams is created, (4) each person in the team has a role, (5) training and games are recorded, and (6) there is a celebration in times of victory (Carlson and Hastie, 1997). As a result, the SE learning environment can help teachers promote students’ motivation because students have social opportunities, make decisions, and enjoy competition, these are very valuable conditions in terms of effort levels and improve children’s motor competence by increasing children’s physical activity (Carlson and Hastie, 1997).

In recent years, studies have introduced TGFU and SE games as useful exercise programs (Healey and Mendelsohn, 2019). However, studies show that both TGFU and SE methods have advantages and disadvantages. For example, TGfU and SE share several aims, concepts, and educational processes. In addition, learning in these models is based on constructivist theories of learning (Dyson et al., 2004).

However, there are also differences between the two models. For example, while SE focuses on a formal and developing sports experience in which students play roles other than players, TGFU focuses on developing the communicative aspects of techniques and tactics and designing assignments accordingly (Casey, 2012). For this reason, each model has limitations when used separately (Hastie and Curtner-Smith, 2006). Nevertheless, a combined TGfU/SE model may yield higher-quality academic and behavioral outcomes (Hastie and Curtner-Smith, 2006). In support of this issue, recently, studies have shown that the combination of TGFU and SE has a greater effect on variables such as creativity (Davoodi et al., 2021) than when each method of SE or TGFU is presented alone. However, despite the claims of these studies (Ríos et al., 2019), it has not been determined experimentally what effect their combination has on the motor skills of DCD children, which are the basis of success in team sports. Therefore, in this study, we included boys with DCD disorder in four traditional groups (control group), SE, TGFU, and combined for 8 weeks and two sessions per week in futsal team sports interventions to find the best type of training to improve the motor competence/proficiency of DCD children. This study aimed to compare the effect of games for understanding, game training, combined, and linear on motor skills of children with developmental coordination disorder. Therefore, it is assumed that the combined intervention has better effectiveness compared to other interventions for children with developmental coordination disorder.

## 2 Materials and methods

The current research applied an experiment, a pre-test-post-test research design with a control group. The statistical population of this study was all 7-year-old children in Türkiye, Ankara. *A priori* power analysis (α = 0.05, 1-β = 0.80, *f* = 0.40) (Mohammadi Orangi et al., 2021) indicated that at least 76 subjects are required. Therefore, 80 participants were selected for this study.

### 2.1 Participants

All participants of this study were boys and were selected from Ankara schools. The average age of the subjects was 7.37 and their standard deviation was 1.12 (see Table 1). The criteria for entering the study are (1) 7-year-old children with developmental coordination disorder, (2) having physical and mental health based on children’s health records other than DCD, and (3) providing written consent of parents. After the approval of the proposal, the code of ethics^1^ was obtained for this work, and written consent was received from all parents; 1 year, all of them received sports insurance from the authors.

**Table 1:** Demographic characteristics of the participants.

Variable	Total *N* = 80 *M* ± SD	Linear *N* = 20 *M* ± SD	SE *N* = 20 *M* ± SD	TGFU *N* = 20 *M* ± SD	Combined *N* = 20 *M* ± SD
Age	7.37 ± 1.12	7.1 ± 1.25	7.44 ± 1.21	7.17 ± 1.02	7.8 ± 1.03
Weight	21.25 ± 2.16	21.23 ± 2.09	20.04 ± 3.47	22.01 ± 1.54	21.72 ± 1.56
Height	119.77 ± 2.51	118.42 ± 2.67	121.09 ± 1.22	119.44 ± 3.44	120.15 ± 2.74

### 2.2 Measuring tool

Movement Assessment Battery for Children-2 (MABC-2) test was used to diagnose motor coordination disorder. This is an effective tool for diagnosing developmental coordination disorder, which has also been used in previous studies (Caçola et al., 2016; Smits-Engelsman et al., 2020). This test is designed to evaluate the motor perception ability of people aged 3–16 and is a suitable test for diagnosing developmental coordination disorder. In this test, hand dexterity, ball skills, and balance are evaluated. In this test, the cut-off point of less than 5% is considered as disordered people, namely, individuals whose scores are less than or equal to 56 in this test. The MABC-2 test has a reliability and validity above 80% in the original version (Chow and Henderson, 2003; Brown, 2021), and Turkey version (Kerkez, 2013).

BOT-2 Bruininks-Oseretsky Test of Motor Proficiency Ed. 2 (short form) was used to measure motor proficiency. The overall set of this test includes 8 sub-tests (4 sub-tests in the group of gross movements, 3 sub-tests in the group of fine movements, and 1 sub-test of upper body coordination) 46 items including a wide profile of high-quality movement skills from separate measures of gross and fine movement skills. The test set provides a comprehensive index of motor proficiency as well as individual scales of fine and gross motor skills for 4–21-year-olds. The duration of the long form is 45–60 min and the short form is 15–20 min. The short form, which contains 14 items from the full collection, can be used as a quick screening tool. This test has the necessary validity and reliability so that the reliability coefficient of its scores in the examination of motor skills was equal to 90%. The retest reliability coefficient of this test is reported as 0.78 in the long form and 0.86 in the short form. The short form measures an individual’s motor skills in general, and the total score indicates the overall skill including gross and fine skills (Bruininks and Bruininks, 2005). This test has also been used in previous studies to measure motor skills (Köse et al., 2021). For this study, standard scores were considered and the total score was the criterion (Mohammadi Orangi et al., 2018).

### 2.3 Procedure

This study was conducted in 2023 and its process started in June. In the first stage, the proposal of this work was approved by the university and permission to carry out the research was obtained. Then a letter was sent from the university to the Ministry of Education to cooperate with this project. Then schools were selected as available to do this. The MABC-2 test was conducted to select children with developmental coordination disorder by a motor behavior specialist who had experience with this test. Then 96 individuals from 15 schools were identified as children with DCD. Of these, 16 individuals were excluded due to reasons such as lack of parental consent for the intervention, having other disorders such as hyperactivity, etc., and finally, 80 male students were randomly divided by one of the researchers into four linear groups of 20 individuals (control), SE, TGFU and combined.

Then each group received futsal training from their coach who had experience in each of the methods. The groups were trained outside the school environment and in a predetermined hall for the children of this study. In this regard, briefing sessions were also held for the trainers before and during the training, and the authors went to the training sessions on a regular basis to ensure that the training was going according to the purpose of the study. These interventions were held as 16 sessions in 8 weeks (see Table 2) and the pre-test and post-test were taken by different people. These people were motor development experts who were completely familiar with how to evaluate motor skills with the BOT-2 test.

**Table 2:** General forms of education (adapted from Davoodi et al. (2021), CC BY-NC 4.0).

Weeks	1-LP	2-TGFU	3-SE	4-Combined
1	Introduction of skills	Choosing the easiest skill	Determining the role of subjects	Getting to know and choosing a simple skill and giving a role to the subjects
2	Presentation of the patern	Designing a group game to learn the selected skill	Selection of skills based on the role of subjects	Game design based on the role of subjects
3	Pattern repetition by subjects	Adjusting the game based on the strengths and weaknesses of the subjects	Setting short-term and long-term goals	Adjusting the game to achieve the set goals
4	Providing feedback to individual subjects	Adding a more complex skill	Offering a reward to children who do the skill correctly	Combining skills and encouraging successful children
5	Changing learned skills based on group average progress	Game design for two combined skills	Changing roles and introducing new skills	Changing roles to suit the combined skills
6	Providing feedback for weaker people to practice more	Introducing a new skill and game design for it	Creating competition between groups	Game in the form of a race to reach the set goal
7	Making the skill harder based on group average progress	Introducing a new skill and game design for it	Changing roles based on changing skills and training for it	Introducing a new skill and giving a role to teach that skill to friends
8	Reducing feedback in proportion to group progress and encourage to complete skills	The combination of the four introduced skills with the designed game	Creating quizzes for all skills learned	Encouraging and rewarding all children and designing a game in which everyone wins, for example, dividing the players into two teams without a goal and the criterion of getting the ball from each other

### 2.4 Intervention

In the traditional training method, each skill was taught separately and feedback was used to improve performance. Children were trained with the teacher’s opinion, and the tasks after learning were taught separately and combined. In this method, finally, after the subjects learned all the materials, the teacher practiced all the tasks together in a game (Crespo et al., 2004; Supriadi, 2019). For example, to practice passing in futsal, the coach would show the skills to the students in the first stage. In the next step, the learners were asked to repeat the pattern. At this stage, the trainer tried to bring the students’ skills closer to the desired pattern with verbal feedback.

In the TGFU teaching method, children learn all the tasks and skills from the beginning in the form of games. In this method, first, simple tasks were practiced in the form of a game, and then skills were integrated in the form of a game so that all the time of the child was spent in the game. In the SE method, competitive games, celebration for victory, giving a role to each of the children, and organizing the goal for the whole exercise, which the children were also aware of, were done. The combined method was the combination of these two methods (Gil-Arias et al., 2017). For example, in the first stage, the trainer classified the skills from simple to complex. For example, if we consider passing, the coach first considered simple passing and designed a game for this skill. So that he prepared balls for the number of players and told the students to face each other pass the ball to each other and change their places quickly. These types of games were varied according to the creativity of the coach and were combined with the progress of the individual. In this method, no model was given and no feedback was used, and whenever the instructor determined that all learners learned the skill (performing the skill with high proficiency), the combination game and complex skills were considered (see Table 2), in which, based on age, each row was trained in two sessions (Gil-Arias et al., 2017).

These exercises were presented for 2 months during 16 sessions and each session lasted one and a half hours. The linear group and SE trained on Saturdays and Mondays from 1:30 to 3 p.m. and from 3 to 5:30 p.m., respectively, and the combined group and TGFU trained at the same time on Sundays and Tuesdays. Finally, the post-test was taken 1 day after the last training session of each group and the results were analyzed.

### 2.5 Statistical method

The demographic information of the subjects was checked and compared by a one-way statistical test. ANOVA test was used for inferential statistics. To check the normality of the data, the Kolmogorov–Smirnov test was used, and before analyzing the data, the hypotheses of the ANOVA statistical test were checked. All statistical works were analyzed in SPSS-24 software at the level of 0.05. Effect sizes smaller than 0.06 were considered small, between 0.06 and 0.14 as moderate, and larger than 0.14 as large (Mohammadi Orangi et al., 2021).

## 3 Results

Kolmogorov–Smirnov test showed that the data are normal at the level of >0.05. The results of the one-way ANOVA test showed no significant difference between the groups in terms of height (*p* = 0.38, *F* = 0.11), weight (*p* = 0.44, *F* = 0.08), and age (*p* = 0.32, *F* = 0.05). Table 3 shows the results of the MABC-2 test. Accordingly, all participants had DCD. Table 4 shows the descriptive information on movement skills in different groups.

**Table 3:** Descriptive statistics related to MABC-2 test.

Group	*N*	*M*	SD
LP	20	36.47	4.25
SE	20	34.52	2.62
TGFU	20	35.35	3.5
Combine	20	37.4	4.43

**Table 4:** Descriptive information of the motor proficiency in the pre-test and post-test of BOT-2 test based on standardized scores.

Variable	Group	*N*	Pre test *M* ± SD	Post test *M* ± SD
Motor proficiency	Linear	20	23.1 ± 2.26	26.75 ± 2.29
	SE	20	22.6 ± 1.72	30.09 ± 2.84
	TGFU	20	23.35 ± 1.71	33.65 ± 2.27
	Combine	20	23.40 ± 2.13	39.7 ± 4.45

The analysis of the covariance method was used to analyze the data and to control the pre-test effect, and the LSD *post hoc* test was used to check the difference between groups. The results are shown in Tables 5, 6. As shown, the effect of the pre-test is not significant, but the effect of the group is significant. In this regard, the results of the follow-up test showed a significant difference between the linear group with SE, linear with TGFU, linear with combination, SE with TGFU, SE with combination, and TGFU with the combination (*p* < 0.001), and according to the descriptive information in Table 4, the experimental group that combined TGFU and SE teaching methods have the greatest effect on motor skills, and then TGFU, SE, and LP groups are listed, respectively.

**Table 5:** The results of covariance analysis for the motor proficiency post-test.

Variable	Test	Mean square	DF	*F*	*p*-value	Eta	Statistical power
Motor proficiency	Pre test Group	0.144 584.75	1 1	0.015 58.93	0.904 0.002	0.001 0.702	0.89 0.104
	Error	9.922	0.75				

**Table 6:** Result of LSD.

Group (I)	Group (J)	Mean difference (I-J)	SD	*p*-value
LP	SE TGFU Combine	−4.16 −6.89 −12.94	1 1 1	<0.001 <0.001 <0.001
SE	TGFU Combine	−2.73 −8.78	1 1	<0.001 <0.001
TGFU	Combine	−6.04	1	<0.001

## 4 Discussion

This study aimed to compare the effect of games for understanding, game training, combined, and linear on motor skills of children with developmental coordination disorder. The results of this study showed that the combined method of SE and TGFU was better than other groups in the post-test. This is even though at the initial test, no significant differences were observed between the 4 investigated groups (3 experimental and 1 control) in terms of motor proficiency. Finally, although there was an improvement in all groups from pre-test to post-test. However, this improvement has been more in the combined group. However, by the standard table of the BOT-2 test (Bruininks and Bruininks, 2005), the post-test scores of all four groups are lower than the fifth percentile rank, which shows that they still have developmental coordination disorder. Nevertheless, the combined group has a better and above average condition considering people with developmental coordination disorder. Based on the authors’ information, no study has been conducted on the effect of TGFU, combined, SE, and linear methods on the motor skills of DCD children; nonetheless, the study of Davoodi et al. (2021) considers TGFU studies a suitable method in education.

For example, Norouzi Seyed Hoseini and Seyed Hossieni (2017) considered TGFU better than the linear method for learning the volleyball serve and Santos et al. (2017) introduced playing games as an effective method for improving creativity. Davoodi et al. (2021) and Davoodi et al. (2023) also considered the combined method (SE, TGFU) and TGFU as a suitable method for creativity and motor skills. However, in the field of motor skills of DCD children, the present study is the first study to be conducted.

To explain the results of this study, it can be said that in the SE method, some advantages such as giving a role to the child and celebrating the victory can be decisive in the child’s independence and self-confidence (Raiola, 2017). However, this method may be harmful at times when the child does not have much role in the game (Gil-Arias et al., 2017). The researchers emphasize that victories and roles in this method should be periodic so that all children can participate in it (Gil-Arias et al., 2017). Nevertheless, the main issue is for meetings where the child does not play a role, and because the world of childhood has its own rules and children are living, these moments of transition can be effective for their learning and growth (Raiola, 2017). Therefore, combining the SE method with a method that emphasizes games can be helpful (Gil-Arias et al., 2017). Combining the TGFU method with the SE method is helpful for children. Because children need to enjoy training. Therefore, behavioral science scientists emphasize that the training environment should be happy and satisfying for children to enjoy it (Dyson et al., 2004). In addition, the type of practice should be such that the child is not judged by other learners and only enjoys the practice. One of the effective ways to implement this is the use of games. In the games, in addition to the fact that the child is involved in the game and does not feel the passage of time, he does not care about the performance of others and is only playing (Dyson et al., 2004). In this case, the child is immersed in the activity, and based on Stodden et al. (2008) model, this activity improves motor skills in the person. When a person’s motor skill improves, he finds himself competent and this increases his motivation to continue the activity. This is an issue that Harter (2000) has also mentioned. Thus, the increase in motor competence increases the self-esteem in the individual and this itself causes the child to continue the activity (Harter, 2000). Therefore, the game helps the child to improve his self-esteem by improving motor skills. This issue is also evident in this study and it was shown that the combined method is the best compared to other methods. Combining games as an important activity in childhood with SE education methods that emphasize improving the child’s self-esteem is a method that can solve the movement competence that is the main problem of DCD children and lead them to continue physical activity. In general, the results of this study showed that childhood is formed by games and games shape children’s world, however, combining games with methods such as SE has better results.

The strength of this study was the comparison of TGFU, combined, SE, and linear methods in DCD children; previous studies either compared only one of these methods with the linear method or focused on normal children. The main limitation of this study is the short duration of the interventions. In addition, the interventions of this study were not standardized and the educators had freedom of action for the interventions. Also, different people have carried out initial and final evaluations using BOT-2. However, these people were specialists who were completely familiar with how to evaluate motor skills with the BOT-2 test. Finally, daily variations in children’s mental and physical state may have (also) contributed to the potentially positive changes in motor skills. Therefore, it is suggested to carry out similar research in the future considering the limitations of the study and the retention test.

## 5 Conclusion

In general, any method that is associated with the game can be suitable for improving motor proficiency. Children must play an active role in training and playing. Therefore, the combination of TGFU and SE method has the characteristics of good exercise and helps the child to improve his motor proficiency. Based on the results of this study, the combination of the TGFU and SE teaching method is the best method for improving the motor proficiency of DCD children compared to TGFU, SE, and linear methods.

## Data availability statement

The original contributions presented in the study are included in the article/supplementary material, further inquiries can be directed to the corresponding author.

## Ethics statement

The studies involving humans were approved by the Middle East Technical University Human Research Ethics committee. The studies were conducted in accordance with the local legislation and institutional requirements. Written informed consent for participation was not required from the participants or the participants' legal guardians/next of kin in accordance with the national legislation and institutional requirements.

## Author contributions

BG: Conceptualization, Data curation, Formal analysis, Funding acquisition, Investigation, Methodology, Project administration, Resources, Software, Supervision, Validation, Visualization, Writing – original draft, Writing – review & editing. SK: Conceptualization, Data curation, Formal analysis, Funding acquisition, Investigation, Methodology, Project administration, Resources, Software, Supervision, Validation, Visualization, Writing – original draft, Writing – review & editing. GB: Conceptualization, Data curation, Formal analysis, Funding acquisition, Investigation, Methodology, Project administration, Resources, Software, Supervision, Validation, Visualization, Writing – original draft, Writing – review & editing.

## Footnotes

^1^0205-ODTUİAEK*2023.
